# Intermetallic compounds in 3D integrated circuits technology: a brief review

**DOI:** 10.1080/14686996.2017.1364975

**Published:** 2017-09-28

**Authors:** Syahira Annuar, Reza Mahmoodian, Mohd Hamdi, King-Ning Tu

**Affiliations:** ^a^ Centre for advanced manufacturing and materials processing (AMMP), Department of Mechanical Engineering, Faculty of Engineering, University of Malaya, Kuala Lumpur, Malaysia; ^b^ Department of Materials Science and Engineering, University of California at Los Angeles, Los Angeles, CA, USA; ^c^ Department of Materials Science and Engineering, National Chiao Tung University, Hsinchu, Taiwan, People’s Republic of China; ^d^ Department of Research and Development, Azarin Kar IND. Co, Kerman, Iran

**Keywords:** IMCs, 3D-ICs, microbumps, low-volume solder microbumps, Pb-free solder joint, 40 Optical, magnetic and electronic device materials, 106 Metallic materials, 201 Electronics / Semiconductor / TCOs, 302 Crystallization / Heat treatment / Crystal growth, 501 Chemical analyses

## Abstract

The high performance and downsizing technology of three-dimensional integrated circuits (3D-ICs) for mobile consumer electronic products have gained much attention in the microelectronics industry. This has been driven by the utilization of chip stacking by through-Si-via and solder microbumps. Pb-free solder microbumps are intended to replace conventional Pb-containing solder joints due to the rising awareness of environmental preservation. The use of low-volume solder microbumps has led to crucial constraints that cause several reliability issues, including excessive intermetallic compounds (IMCs) formation and solder microbump embrittlement due to IMCs growth. This article reviews technologies related to 3D-ICs, IMCs formation mechanisms and reliability issues concerning IMCs with Pb-free solder microbumps. Finally, future outlook on the potential growth of research in this area is discussed.

## Graphical abstract

**Figure:**
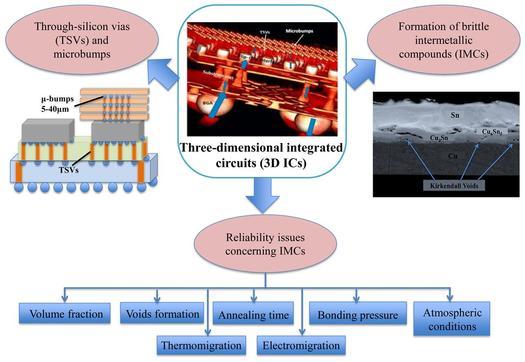

## Introduction

In the past several decades, the semiconductor industry has seen the integration of many electronic components such as resistors, transistors and capacitors onto silicon chips, creating so-called two-dimensional integrated circuits (2D ICs). The exponential growth in the number of transistors per unit area or density of circuits per chip has been in line with Moore’s law prediction. However, Moore’s law is momentarily near the end of its prediction due to physical limitations as well as economic constraints. To overcome these problems, the microelectronics industry is attempting to combine chip and packaging technologies by stacking chips vertically. Vertical stacking was introduced in the late 1990s by Matsumoto et al. [1]. They demonstrated a permanent wafer bonding technology with polycrystalline through-silicon via (TSV), namely the via-first approach for 3D ICs. This was followed by Ramm et al. [2], who proposed a temporary wafer bonding technology with W-TSVs that combines the via-middle and via-last methodologies.

The accessible manufacturing approaches of 3D ICs by different design methods may have led to the integration of more electronic functionalities at shorter interconnect lengths [3–5]. At the recent manufacturing level, 3D ICs consist of several layers of interconnected chips that are built on top of the Si chips. This is achieved by utilizing low joint volumes and thicknesses in the form of microbumps [6–8]. This type of joint technology has rapidly grown in demand with great engineering efforts for vertical interconnects in conjunction with through-silicon via (TSV) [9–13]. Thousands of microbumps solder joints are normally supported with two other types of joints, namely ball grid array (BGA) and flip-chip C-4 (or controlled collapse chip connection) solder joints to constitute the 3D ICs [14].

The shift to lead (Pb)-free solder in solder microbumps is mainly intended to replace Pb-based solder that causes huge environmental and toxicity concerns [15,16]. The selection of Pb-free solder is most often configured by Sn-based alloys such as Sn-Ag-Cu (SAC), Sn-Cu and Sn-Zn, which reveal good solder wettability and ultimately form a rod-type structure [17,18]. However, in solder microbumps, the high diffusivity and quick reaction of Pb-free solder together with the shrinking chip dimensions offered by 3D ICs cause extensive formation of intermetallic compounds (IMCs) in the solder microbumps [19,20]. Eventually, the excess IMCs appear brittle, particularly under drop conditions [21]. Several studies have presented a clear failure mode change from ductile in the bulk solder to brittle in the IMCs layer when the parameters of aging time, deformation speed and IMCs layer thickness are increased [22–24]. Lee et al. [25] stated that the mechanical properties of a solder joint are dominated by the properties of the IMCs phase. Therefore, this paper presents a review of recent technologies comprising 3D ICs, namely TSV and microbumps, followed by IMCs mechanism formation and, lastly, reliability issues of microbumps concerning IMCs.

## Three-dimensional integrated circuits: the technology

**Figure 1.**
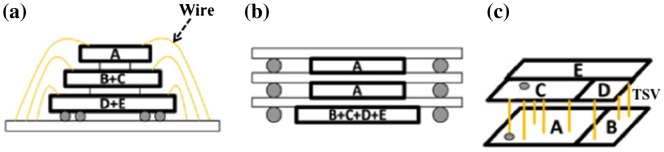
Schematic comparison of three-dimensional (3D) interconnections of (a) stacked die package by wire bonding; (b) package on package by ball grid array (BGA); and (c) wafer and/or dies interconnected with through-silicon via (TSV) [28].

Monolithic three-dimensional integration (M3DI) technology is a scheme that enables 2D ICs to have multiple stacking device structures connected at the transistor level by robust electronic design automation (EDA) tools [26,27]. Figure 1 shows a schematic comparison of die-level or wafer-level three-dimensional interconnections that are distinguishable from one another.

Compared with off-chip signalling 3D packaging, 3D ICs interact through on-chip signalling [28,29]. In 3D ICs, the most significant elements of the enabling technology for handling and double-sided processing of extremely thin chips are the temporary bonding and debonding processes at lower temperature and higher throughput [30]. A well-known bonding method that has become noteworthy in the production process for 3D ICs stacking minimization is thermal compression bonding, whereby two metals are ideally bonded together by heating and compression [31,32]. The temperature for bonding requires that material diffusivity between two metal substrates and the solder be taken into account [33]. Wu and Kumar [34] mentioned that the capability of lower temperature bonding is important for relaxing the thermal stress induced by the coefficient of thermal expansion mismatch between the copper solder and silicon wafer. In low-temperature bonding, a sufficient amount of bonding materials in conjunction with suitable surface modifications between the upper and lower dies may assist the reaction phase formation and progression. For instance, to achieve transient liquid phase (TLP) bonding at less than 300 °C, utilizing molten gallium solder-platinum under bump metallurgy (UBM) triggers a liquid-solid reaction in the microbumps’ metallurgical structure bonding. Meanwhile, modifying the argon plasma-treated metal surface can lead to even lower temperature bonding (e.g. 200 °C) due to the solid state bonding reaction resulting from low surface roughness and a defect-free interface [35–37].

### Through-silicon via (TSV)

In 3D ICs technology, the TSV interconnection in chip stacking is introduced to provide connectivity between different designed parts for proper signal propagation and delivery, especially in high bandwidth and high-density dynamic random access memory (DRAM) [38–42]. Sukharev [43] and Lau [44] reported finite element analysis simulations of TSV upon electromigration (EM) failure, which is when the TSV is unable to deliver the necessary voltage to any circuitry gate. Ryu et al. [45] and Stiebing et al. [46] suggested considering thermal cycling, which causes the TSV to extrude from the surrounding matrix or substrate. This phenomenon occurs due to a coefficient of thermal expansion mismatch between the surrounding substrate and the metal filler in TSV. Roh et al. [47] overcame these reliability issues by alloying copper (Cu) together with tungsten (W) metal filler. It was observed that Cu-W alloy fills the TSV-Si substrate and exhibits no appreciable delamination compared to a single Cu-filled TSV-Si substrate.

### Microbumps

**Figure 2.**
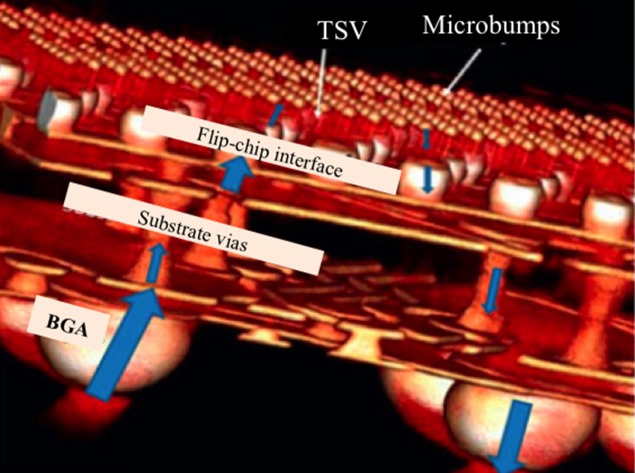
Synchrotron radiation tomography of 3D ICs test sample with the blue arrows representing the electrical flow during device operation [48].

A common 3-level solder joint packaging structure in 3D ICs is revealed in the three-dimensional view in Figure 2. The 3-level joint is positioned with a ball grid array (BGA) in the bottom row, controlled collapse chip connection (C4) flip-chip solder joints in the middle, and microbumps at the top between two silicon chips [48]. The main purpose of solder joints in microelectronic devices is to provide good metallic bonding [49,50].

Microbumps are desirable for joining owing to their capability to increase the circuit density from the substrate sides to several miniaturization chips. The most well-known method of preparing microbumps is the soldering process [51]. In this process, microbumps can be obtained in sizes ranging from 10 μm to 30 μm [52–54]. In order to continuously scale down the microbumps size to 1 μm, the electroplating method is employed, which is subsequently followed by patterning the pillar-type structure of the microbumps [55]. Talebanpour et al. [11] suggested sandwiching electroplated microbumps between micron-size dies, while substrates are aimed to increase the aspect ratio of the solder joint. The intention is to mainly replace higher volume solder joints such as the flip-chip and lessen the requirement for expensive substrate design features [25,56,57]. The post-prepared microbumps will undergo a reflow process at temperatures above the melting point of the solder and below the melting point of UBM or metal substrate. This process generally gives the solder microbumps a round shape, resulting in high-yield joining and superior electrical properties compared with non-reflowed microbumps [50,58,59].

Since the size of microbumps has been progressively scaled down, the remaining challenge is mainly in the ability of the microbumps to preserve their mechanical stability whilst maintaining their function. The requirement to utilize underfill is approached for mechanical stability, and to enhance joint strength between stacked chips and prevent microbump corrosion [60]. There are several methods of injecting underfill in the ultra-narrow gaps between the stacked chips. Fukushima et al. [4] introduced microbump bonding through nonconductive film (NCF) to allow complete, void-less adhesive filler into the extremely narrow gaps between the chips and wafer by capillary force. The difficulty with underfill flowing by capillary force is the potential need for high external thermal compression to flatten the thin dies, which would then lead to additional mechanical reliabilities [61]. Recently, Ohyama et al. [62] mentioned that pre-applied fully coated underfill before bump bonding was already established in the 1990s, whereby no-flow of underfill was intended to attain both underfill and bump interconnection. However, underfill entrapment caused by both capillary force and pre-applied underfill may influence microbumps reliability. Liu et al. [63] emphasized that there must not be any filler trapped in the extrusion of the compressed microbumps, otherwise they will exhibit poor electrical conduction. Ohyama et al. [62] overcame these challenges by employing a hybrid bonding process, whereby the excess pre-applied underfill-coated microbumps are removed by polishing. The consequent several nanometres of the upper and lower half of the microbump tip can thus be perfectly bonded with no underfill entrapment.

## IMCs formation

As extensions from the microbumps structure, the inevitable growth of intermetallic compounds (IMCs) between the solder and substrate can hugely influence the reliability of smaller solder sizes in 3D ICs [64–66]. It is noted that the most popular metallurgy configurations of Pb-free solder microbumps are from copper (Cu) and tin (Sn)-based alloy with more than 97wt% Sn metal. The interdiffusion of this binary alloy is interestingly favoured due to the anisotropic properties offered by the tetragonal β-Sn phase [67]. In general, the diffusion of metal atoms into the metallic semiconducting phase of β-Sn may differentiate the diffusion energy of the metal to be penetrated with respect to different crystallographic orientations governed by the β-Sn crystal structure [68,69]. In terms of adsorption energy (*E*_ad_) and penetration energy (*E*_pe_), Table 1 reveals that various M atoms are strongly adsorbed onto the surface and subsequently penetrate faster into the *c*-axis than the *a*-axis of Sn [69].

**Table 1.** Different adsorption and penetration energies of M atoms on the *a*-axis and *c*-axis of Sn solder [69].

M atoms	*E*_ad_ /(eV)		*E*_pe_/(eV)	
	*a*-axis	*c*-axis	*a*-axis	*c*-axis
Ni	−4.84	−5.18	0.57	0.06
Cu	−3.05	−3.58	0.61	0.18
Ag	−1.96	−2.65	0.78	0.43
Au	−2.11	−2.77	0.79	0.49
In	−1.27	−2.88	0.88	0.57

**Figure 3.**
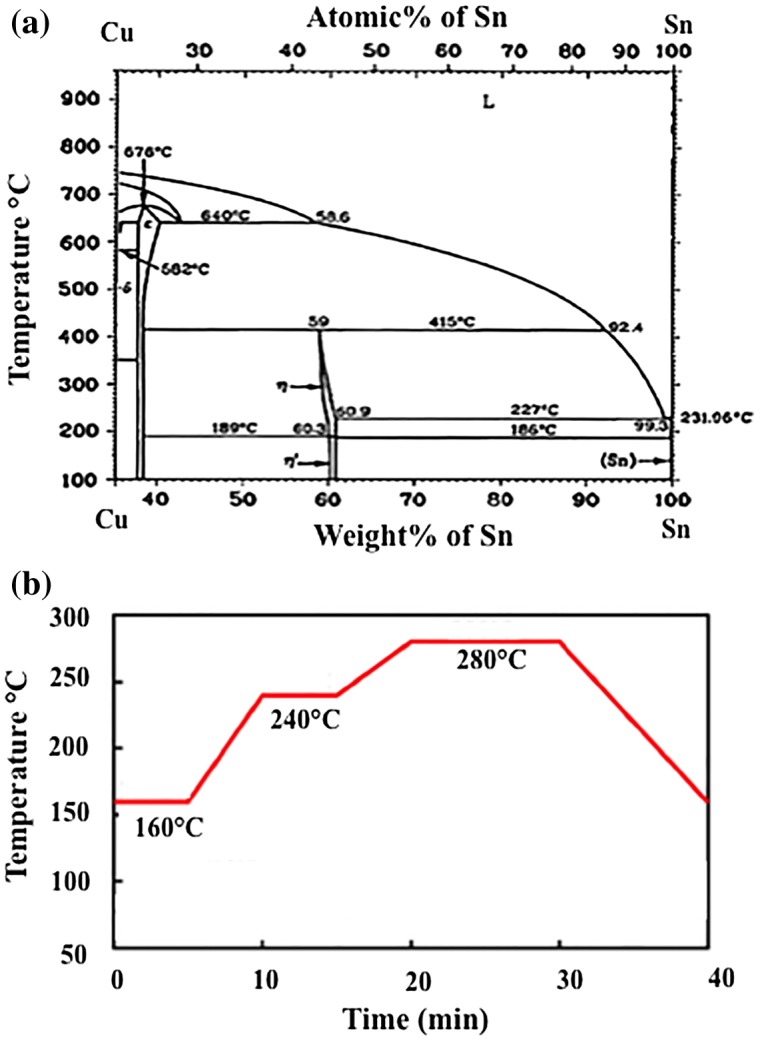
(a) Phase diagram [74] and (b) bonding profile of binary Cu-Sn couple [75].

In conjunction with this metallurgy, the IMCs formation of Cu and Sn binary systems has been increasingly and widely discussed [70–72]. In order to successfully assemble a Cu and Sn binary system, a phase diagram and temperature profile of this binary alloy are necessary to understand the phase evolution resulting from their interactions (Figure 3) [73].

In the Cu-Sn system, two types of IMCs layers are normally present, namely η-Cu_6_Sn_5_ or Cu_6_Sn_5_ and ε-Cu_3_Sn or Cu_3_Sn. A number of intricate studies have been conducted to understand the microstructure of these IMCs [76,77]. The IMCs configuration of soldered microbumps usually involves heating, reflow and solidification [78,79]. During the early formation of Cu_6_Sn_5_ IMCs a liquid-state reaction takes place, whereby the molten Sn from the solder reacts directly with the Cu substrate, as per Equation (1).(1)
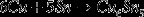



This is followed by a reflow process that facilitates a reaction between some remaining Sn atoms from soldering and the Cu_6_Sn_5_ interface formed earlier [79]. As the process continues to progress, the subsequent solid-state reaction of Cu_6_Sn_5_ IMCs requires the diffusion of Cu atoms within Cu_6_Sn_5_ grains from small to large grains due to the distinct concentration gradient and different radii of Cu atoms. This phenomenon is known as Ostwald ripening [80]. The Cu diffuses from smaller to larger grains, thus concurrently increasing the large Cu_6_Sn_5_ grains and shrinking the small grains. The diffusion coefficient, *D*, is expressed as the Arrhenius Equation (2):(2)
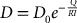



where 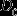 is a pre-factor, *Q* is the activation energy for diffusion, *T* is the temperature and *R* is the gas constant (8.314 J/K mol). The growth of Cu_6_Sn_5_ is enhanced during solidification, because the super-saturated Cu atoms in the molten solder are rejected and precipitated on the existing Cu_6_Sn_5_ [81]. The morphological transition of IMCs can be observed clearly in the solidification pattern, either in the eutectic or peritectic transformation, which denotes a stable solid-solid and solid-liquid equilibrium reaction [82].

Likewise, thermal solid state aging causes a transformation from existing low-temperature Cu_6_Sn_5_ IMCs to existing high-temperature Cu_3_Sn IMCs [11]. Ko et al. [83] indicated that Cu_3_Sn arises largely from the consumption of Cu_6_Sn_5_, as in Equation (3):(3)
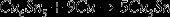



As the temperature increases, the extra energy gained by the Cu atoms at the substrate interface causes them to continue to react with pre-existing Cu_6_Sn_5_ until it grows into a thick layer and becomes a diffusion barrier to the Cu diffusion [84]. This blocks the diffusion channel to the Cu_6_Sn_5_ layer and a Cu_3_Sn layer grows beneath it simultaneously [85]. Therefore, the Cu_3_Sn layer is sustained between the Cu substrate and Cu_6_Sn_5_ IMC layer. The addition of a Ni layer in the Cu-Sn system may act as a diffusion barrier where no further Cu_3_Sn formation can occur [86]. Temperature influences both the morphological shape and structure of the IMCs established at the same time. At low temperature (50 °C), the scallop-type Cu_6_Sn_5_ layer transforms into planar-like Cu_6_Sn_5_ until the temperature increases up to 150 °C, when it eventually grows into a uniform Cu_3_Sn layer [87]. In the case of Ni-Sn solder, the large and round grains of Ni_3_Sn_4_ IMCs fully transform into whisker-type, coarsened grains when the annealing temperature is increased from 235 °C to 290 °C [88].

## Reliability issues concerning IMCs

### Volume fraction

The brittle failure of IMCs, which are susceptible to concentrated stress, may cause structural defects in the microbumps [89]. Therefore, it is vital to surpass the mechanical strength of the IMCs. The fracture toughness of a microbumps’ joint as a function of the volume fraction of IMCs was investigated by Talebanpour and Dutta [90]. They have stated that the fracture toughness, G_C_, of the microbumps decreases with increasing IMC volume fraction, *h*_IMC tot_/*h*_jt_ (total percentage of IMCs/strain rate). The hard constituents of IMCs replace the soft phase in the microbumps, and therefore, the increased IMC content enhances the apparent flow stress of the thinner solder joint. Joints with lower solder percentage can fracture much more easily than joints with greater solder content. This means that less plastic work dissipates in joints with lower solder content.

### Void formation

**Figure 4.**
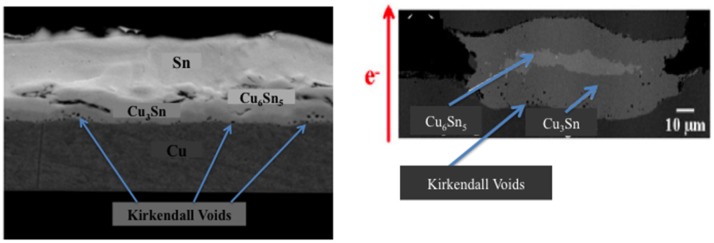
Scanning electron microscopy (SEM) images of Cu/Sn diffusion coupled with Kirkendall void formation at the Cu_3_Sn layer (a) without electromigration effect [76] and (b) with electromigration effect [95].

The presence of voids in IMC structures is unavoidable as a result of atom migration upon reaching different thermal states. In IMCs established at low temperature, microvoids usually appear in the middle of the solder joint, due to molar volume changes resulting from the growth of Cu_6_Sn_5_ IMCs in the Sn solder [91]. Meanwhile, in IMCs established at high temperature, numerous Kirkendall voids are produced due to the high-energy metal within the IMCs that triggers flow, leaving empty lattices behind. Minho et al. [76] demonstrated Kirkendall voids in the Cu_3_Sn layer during annealing at high temperature of ~200 °C for 1 h (Figure 4(a)). In an electromigration test, the Kirkendall voids in Figure 4(b) may evoke current crowding, thus accelerating void propagation during current stressing [92]. Consequently, the presence of voids enclosed in the IMCs will seriously affect the reliability of the microbumps [93,94].

### Annealing time

**Figure 5.**
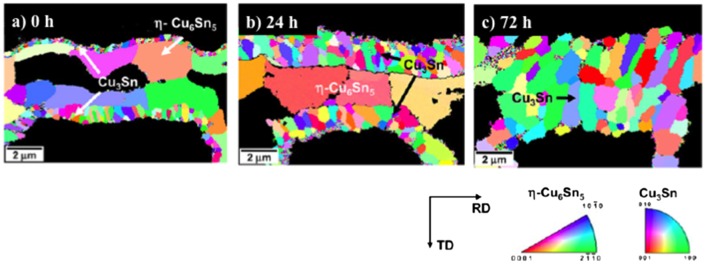
EBSD orientation maps (TD) of Cu-Sn IMCs (RD: reverse direction and TD: transverse direction) [96].

The behaviour and thickness of IMCs structure varies with increasing annealing time. It appears that both Cu_6_Sn_5_ and Cu_3_Sn IMCs follow a certain crystallographic orientation. As shown in the electron backscatter diffraction (EBSD) orientation maps in Figure 5, no preferred crystallographic orientation is found in the pre-existing Cu_6_Sn_5_ layer in the as-soldered state. After 24 h of annealing, the Cu_6_Sn_5_ layer has a dominantly favoured (0001) orientation [96] and by further increasing the annealing time to 72 h, a Cu_3_Sn layer forms, which follows a (100) orientation.

The total thickness of both Cu_6_Sn_5_ and Cu_3_Sn IMCs increases with increasing annealing time while other parameters are kept constant (Table 2) [97,98]. This is in agreement with the empirical power law relationship in Equation (4) [99].(4)
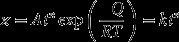



**Table 2.** Analysis of total IMC thickness with respect to different aging times, constant atmospheric condition and constant temperature for each solder joint.

Solder joint	Atmospheric condition	Annealing temperature (°C)	Aging time(hours)	Total thickness of IMCs (μm)(Cu_6_Sn_5_+ Cu_3_Sn)	Ref.
Sn3.5-Ag	Air	260	0.16	20.50	[100]
			0.50	29.30	
			1.00	38.45	
Sn0.7-Cu	Vacuum	240	24.0	3.77	[101]
			72.0	4.40	
			120.0	4.85	
Sn3.0-Ag0.5-Cu	Air	150	100.0	4.80	[102]
			200.0	5.50	
			500.0	6.50	

where 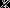 indicates the IMCs layer thickness depending on time *t*; *A* is a growth constant; *n* is the time exponent; *Q* is the activation energy; *R* is the gas constant (8.314 J/K mol); *T* is the absolute temperature and *k* is the growth rate constant. This relationship can execute the mechanisms involved in IMCs growth, as determined by the time exponent, *n*. If *n* is 1, it indicates that IMCs growth is governed by the reaction rate. If *n* is 2, IMCs growth is influenced by the diffusion of reaction elements present, and if *n* is close to 3, growth is affected by a diffusion-controlled mechanism with coarsened IMC grains [103].

### Bonding pressure

Several research works have been carried out on applying distinct pressure in microbumps bonding. The different pressures applied promote varying microstructure growth and IMCs joint status while keeping other parameters, such as time and temperature, constant. At low soldering pressure, some areas of the microbumps may be disconnected and in loose contact with the desired position. This condition may cause smaller amounts of flowing metal solder during bonding, thus exhibiting a different microstructure evolution than under high soldering pressure on the final IMCs microstructure [104]. The fracture behaviour of microbumps as a function of IMCs varies with the different bonding forces applied. The location of the shear stress applied changes from the Cu_6_Sn_5_ IMCs/solder bump interface to the UBM/Cu pillar bump interface, which implies that the fracture modes change as the bonding force increases [105]. The fracture in the solder bump matrix is first ductile and then it transforms into a brittle mode upon reaching the IMCs area [11]. The fracture modes fall into three categories: (1) fractures within the solder bump matrix, (2) interfacial fractures at the Cu_6_Sn_5_ IMCs/solder bump interface, and (3) interfacial fractures at the UBM/Cu pillar bump interface [105]. Debonding or fracturing occurs at the lowest bonding point of the Cu_6_Sn_5_ IMCs/solder bump.

### Atmospheric conditions

Exposing microbumps to different atmospheric conditions will eventually affect their reliability, which is evaluated in terms of the IMCs’ microstructure. Regarding microbump bonding exposed to the oxygen atmosphere, Panchenko et al. [106] found that the most challenging issue is pore progression due to the presence of oxygen from the atmosphere. This may lead to the formation of large and hard-to-eliminate voids in the final IMCs structure. Interestingly, in microbump bonding that takes place in both nitrogen and formic acid atmospheres, much looser final IMC particles coalesce in nitrogen than in formic acid atmosphere. Consequently, the shear strength of the joint in a nitrogen atmosphere is weaker [107].

### Electromigration (EM)

**Figure 6.**
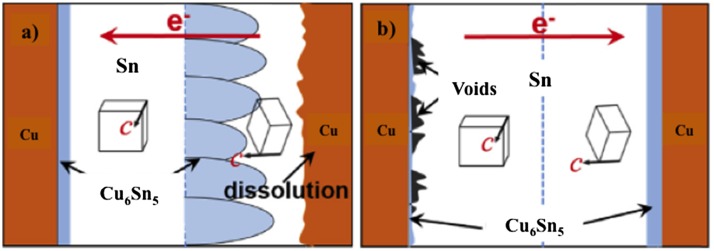
Schematic diagram of two *β*-Sn grain interconnects separated by grain boundaries with different electron flows: (a) forward direction and (b) reverse direction [112].

Among the unavoidable factors that influence microbumps reliability is electromigration (EM), which is defined as enhanced atomic diffusion driven by high electric current density [108]. This phenomenon may induce microbumps failure because EM preferentially flows according to a specific solder material orientation, especially in a Sn-based metal alloy. For instance, the Cu-Sn binary system in Figure 6 represents a schematic diagram of the anisotropic Sn grains with respect to different electron flow directions. The figure suggests that if the *c*-axis of β-Sn and the electron flow direction are almost parallel, Cu atoms from the substrate will largely tend to diffuse into the Sn and lead to its dissolution. However, it is unlikely that a different diffusion mechanism governs when the electron flow is the reverse and perpendicular to the direction of the *c*-axis in β-Sn. EM can seriously affect the microstructure evolution mechanism of IMCs, together with voids formation and propagation under current stressing time [109]. On the other hand, since in the EM tests the electron flow from the cathode to the anode, it is important to decide on what materials to select for each respective electrode**.** This is because, in the EM test, a notable polarity effect of the asymmetric thickness of IMCs is observed due to the enhanced transport material. Eventually, IMCs thickness will be dependent on different electron flow directions [108]. Kim et al. [110] and Tian et al. [111] reported that before the microbumps-IMCs are fully transformed, the previously existing Cu_6_Sn_5_ layer at the cathode side decreases due to current stressing from the cathode to the anode and the limited supply of Sn solder atoms. At the same time, the Cu_3_Sn layer at the anode side keeps increasing due to the continuous proliferation of a Cu substrate according to the atomic flux effect.

### Thermomigration

Correlated to EM, thermomigration (TM) is a phenomenon that involves heat generation and dissipation over numerous chips stacked in 3D ICs. TM explains the atomic diffusion from high-temperature to low-temperature regions, which can cause atom migration problems in solder microbumps structures [3,110,113,114]. The temperature gradient exists since the microbumps undergo thermal compression bonding and reflow as a function of different thermal conductivities of the substrate, chips and solder [115]. Table 3 summarizes the asymmetrical growth of IMCs, whereby Cu atoms are migrating from the hot to the cold side [116].

**Table 3.** Asymmetric growth of IMCs on the cold and hot sides.

Different sides	Growth of IMCs	Growth mechanism of IMCs	Morphology changes	Typical explanation
Cold side	Cu_6_Sn_5_ is enhanced	Reaction and thermo- migration-controlled	Scallop-like Cu_6_Sn_5_ transformed into layer-like	- Sufficient Cu atomic flux on the cold side
Hot side	Cu_6_Sn_5_ and Cu_3_Sn are hindered	Grain boundary and thermo- migration-controlled	Scallop-like Cu_6_Sn_5_ is maintained	- Insufficient Cu atomic flux on the hot side

**Figure 7.**
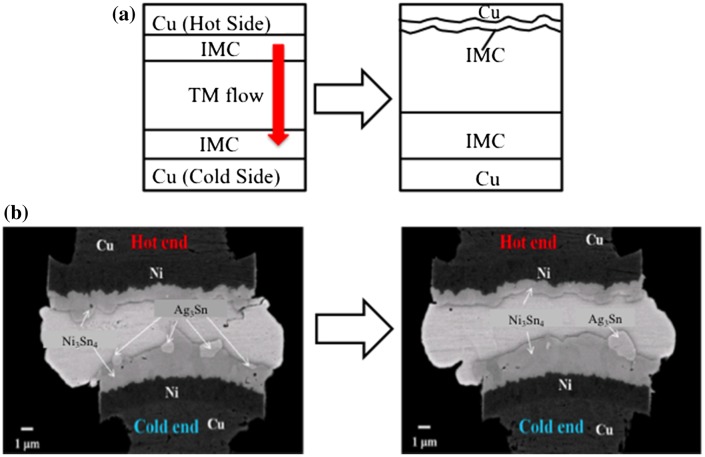
(a) Schematic illustration of the asymmetric growth of IMCs during thermomigration and (b) SEM images of the asymmetric growth of Ni_3_Sn_4_ IMCs under thermomigration conditions at the hot end (190°C) and cold end (100°C) for 150 h [113].

Atomic diffusion is regulated by the temperature gradient in TM and is assisted by the grain orientation of metal microbumps, especially in the Sn-based metal alloy configuration [117]. A large atomic flux is induced from the hot to the cold end if *θ*, the angle between the *c*-axis of β-Sn and the temperature gradient, is small. Thus, asymmetric growth of IMCs as well as Cu substrate dissolution at the hot side and large IMCs growth at the cold side will occur, as illustrated in Figure 7(a). In Figure 7(b), the Sn-Ag solder with a Ni layer on the Cu substrate displays asymmetric Ni_3_Sn_4_ IMCs thickness at both hot and cold ends. The IMCs layer appears thicker at the cold end than the hot end [113].

## Summary and future outlook

Research on 3D ICs has been continuously developing over the past decade. With the rapid progress, we are optimistic that the acquired findings and deeper understanding will benefit commercialization through knowledge transfer. The technologies of TSV and solder microbumps in 3D ICs have enabled the desired currently downsized chips to be interconnected with the assistance of two other joints, i.e. ball grid array (BGA) and controlled collapse chip connection (C4). A few useful means of utilizing underfill material to assist with microbumps bonding were listed. In the future, microbumps bonding may evolve to solid state bonding reactions that happen at low temperatures rather than the current liquid-solid bonding reactions that occur at high temperatures.

Intermetallic compounds formation is unavoidable, especially in smaller size joints. It is noted that the most common growth mechanisms of intermetallic compounds are heating, reflow and solidification. Eventually, intermetallic compounds may exhibit both metal and ceramic properties, which are conductive and yet brittle. Despite the ongoing research, a number of important factors – such as low solder volume of microbumps, void formation, annealing time, bonding pressure, atmospheric condition, electromigration and thermomigration – can seriously affect the mechanical reliability of microbumps through the formation of intermetallic compounds.

The symmetric and asymmetric growth of intermetallic compounds is well-understood due to the atomic migration that is affected by both interior and exterior factors acting on the microbumps. The symmetrical growth of intermetallic compounds may be affected by (1) annealing time and (2) temperature regime: heating or cooling. Meanwhile, the asymmetrical growth of intermetallic compounds is caused by the imbalance of (1) electric current flow in the electromigration test and (2) the temperature supplied at one side in the thermomigration test. Therefore, in order to control the formation of intermetallic compounds according to the intended application, it is highly recommended to technically manipulate the above-mentioned factors. It is possible for intermetallic compounds to be beneficial to enhancing the reliability of solder microbumps if the formation of the compounds can be controlled well.

## Disclosure statement

No potential conflict of interest was reported by the authors.
